# PLGA-microspheres-carried circGMCL1 protects against Crohn’s colitis through alleviating NLRP3 inflammasome-induced pyroptosis by promoting autophagy

**DOI:** 10.1038/s41419-022-05226-5

**Published:** 2022-09-10

**Authors:** Jie Zhao, Ye Sun, Haojun Yang, Jun Qian, Yan Zhou, Yu Gong, Yi Dai, Yuwen Jiao, Weiming Zhu, Honggang Wang, Zhiliang Lin, Liming Tang

**Affiliations:** 1 Department of Gastrointestinal Surgery and Central Laboratory, The Affiliated Changzhou No. 2 People’s Hospital of Nanjing Medical University, Changzhou, China; 2 Department of Orthopaedics, The First Affiliated Hospital of Nanjing Medical University, Nanjing, China; 3 Department of Gastrointestinal Surgery, The Affiliated Changzhou No. 2 People’s Hospital of Nanjing Medical University, Changzhou, China; 4 Department of General Surgery, Jinling Hospital, Medical School of Nanjing University, Nanjing, China; 5 Department of General Surgery, Taizhou People’s Hospital, Taizhou Clinical Medical School of Nanjing Medical University, Nanjing, China; 6 Department of Colorectal Disease, Intestinal Microenvironment Treatment Center, Shanghai Tenth People’s Hospital, Tenth People’s Hospital of Tongji University, Shanghai, China

**Keywords:** Crohn's disease, Crohn's disease

## Abstract

This study aimed to at explore exploring the biological functions of dysregulated circRNA in Crohn’s disease (CD) pathogenesis, with the overarching goal of and providing potential novel therapeutic targets. CircRNA microarray and quantitative real time-polymerase chain reaction (qRT-PCR) analyses were performed to investigate and verify the candidate dysregulated circRNA. The Next, clinical, in vivo, and in vitro studies were performed to investigate explore the biological function and mechanisms of the candidate circRNA in CD. The therapeutic effect of poly (lactic-co-glycolic acid)-microspheres (PLGA MSs)-carried oe-circGMCL1 in experimental colitis models of IL-10 knock-out mice was assessed. CircGMCL1 was identified as the candidate circRNA by microarray and qRT-PCR analyses. Results showed that circGMCL1 expression was negatively correlated with CD-associated inflammatory indices, suggesting that it is a CD-associated circRNA. Microarray and bioinformatics analyses identified miR-124-3p and Annexin 7 (ANXA7) as its downstream mechanisms. The in vitro studies revealed that circGMCL1 mediates its effects on autophagy and NLRP3 inflammasome-mediated pyroptosis in epithelial cells through the ceRNA network. Moreover, the in vivo studies identified the therapeutic effect of PLGA MSs-carried oe-circGMCL1 in experimental colitis models. This study suggests that circGMCL1 protects intestinal barrier function against Crohn’s colitis through alleviating NLRP3 inflammasome-mediated epithelial pyroptosis by promoting autophagy through regulating ANXA7 via sponging miR-124-3p. Therefore, circGMCL1 can serve as a potential biological therapeutic target for Crohn’s colitis.

## Introduction

Crohn’s disease (CD), a major type of inflammatory bowel disease (IBD), is a complex chronic disorder which affects the gastrointestinal tract [1]. The pathogenesis of CD is complicated and has not yet been fully elucidated to date [2]. However, accumulating evidence has revealed that immunology, gut microbiota, environment factors, and genetic predisposition are involved in the pathogenesis of CD [3, 4].

Circular RNAs (circRNAs), a newly discovered class of noncoding RNAs (ncRNAs), play important roles in various nervous system disorders [5], cancers [6], and autoimmune disease [7]. The theory of competing endogenous RNAs (ceRNA) is one of the widely recognized and most important mechanism through which circRNAs are involved in the progression of various diseases [8]. In recent years, the association between circRNAs and CD has attracted a lot of attention. A recent study by Ye et al. [9] found that has_circRNA_103765 in peripheral blood mononuclear cells (PBMCs) can serve as a proinflammatory factor by sponging miR-30 family in CD. Xiao et al. [10] also demonstrated that circHIPK3 can promote the intestinal epithelium homeostasis by inhibiting miR-29b in inflammatory bowel disease (IBD).

This study aimed at exploring the biological functions of dysregulated circRNA in CD pathogenesis, with the overarching goal of providing potential novel therapeutic targets. Microarray results identified the most dysregulated circRNA, hsa_circ_0055097 (circGMCL1), in colon tissues, which was further validated by quantitative real time-polymerase chain reaction (qRT-PCR). Moreover, its expression showed a linear negative correlation to CD-associated inflammatory indices, indicating that circGMCL1 is a CD-specific circRNA. Mechanistically, the miR-124-3p/Annexin 7 (ANXA7) pathway was verified as the downstream of circGMCL1. Considering that *ANXA7* is an autophagy-associated gene [11], we functionally validated the inhibitory function of circGMCL1 for NLRP3 inflassome-induced pyroptosis and proinflammatory cytokines by inducing autophagy. In addition, overexpression of circGMCL1 ameliorated experimental colitis and improved intestinal barrier function in IL-10 knock-out (IL-10 KO) mice. In summary, these findings strongly indicate the biological functions of circGMCL1 and its potential as a therapeutic target for CD.

## Materials and methods

### Patient samples

Colon samples were obtained from 78 CD patients who underwent elective colectomy at the Inflammatory Bowel Disease Center of Jinling Hospital between 2018 and 2021. The clinical study was conducted in accordance with the Helsinki Declaration, with ethical approval by the Ethics Committee of Jinling Hospital. After surgical resection, soybean sized specimens containing lamina propria (LP) were collected, placed in liquid nitrogen, and then immediately stored at −80 °C for further analysis. In addition, colonic biopsy specimens were obtained from 78 age- and sex-matched normal controls (NCs) undergoing colonoscopy examination. Inclusion criteria: (1) aged 18–65 years; (2) pathologically diagnosed with colonic or ileocolonic CD; and (3) underwent elective colectomy with signed informed consent. Exclusion criteria: (1) with malignancies; (2) ileal or ileocolonic CD with diffuse small bowel lesions; (3) undergoing emergency operations due to complications (e.g., intestinal perforation, hemorrhage, etc.); (4) with other autoimmune diseases; and (5) declined to participate. CD-associated inflammatory indices, including C-reactive protein (CRP), Crohn’s disease activity index (CDAI) [12], and SES-CD [13], were assessed before surgery. All participants provided written informed consent prior to the study. Table 1 shows the detailed demographic data (age, gender, etc.) for all participants.

**Table 1:** Patient demographics for the cohort.

	Discovery set (microarray)			Validation set (qRT-PCR)		
	CD	NC	*P* value	CD	NC	*P* value
Number	3	3	–	75	75	–
Age (years)	36.7 ± 2.1	37.7 ± 2.5	0.624	38.2 ± 5.1	39.8 ± 5.6	0.597
Gender, *n* (%)	–	–	1.000	–	–	
Male	2	2	–	45	38	–
Female	1	1	–	30	37	–
BMI (kg/m^2^)	21.0 ± 1.9	21.1 ± 2.0	0.762	21.6 ± 2.9	22.3 ± 2.7	0.128
Disease location, *n* (%)	–	–	–	–	–	–
Colonic	2	–	–	20	–	–
Ileocolic	1	–	–	55	–	–

*CD* Crohn’s disease, *NC* normal control.

### Animal experiments

IL-10 KO mice on C57BL/6 background (GemPharmatech Co., Ltd., Nanjing, China) under specific pathogen-free (SPF) conditions were used as the experimental colitis models. Animal experimental procedures were approved by the Laboratory Animal Center, Nantong University (No. S20200323-289).Wild type (WT) and IL-10 KO mice (female, 12-week-old) were randomly assigned into three groups (six mice in each group): (a) WT group; (b) IL-10 KO + oe-circGMCL1+MS group (oe-circGMCL1 i.g., once a day for seven consecutive days), and (c) IL-10 KO + oe-control group (empty PLGA MSs i.g., once a day for seven consecutive days). Disease activity index (DAI) was assessed based on the conditions (ruffled fur, occult fecal blood, and rectal prolapse) described in previous study [14]. Mice were sacrificed one week after drug administration. The entire colons were harvested for length measurement and proximal colon tissues were isolated for subsequent experiments.

### Histological analysis

To evaluate the histological inflammation, the obtained proximal colon sections were stained with hematoxylin and eosin (HE), and Alcian blue-Periodic acid Schiff (AB-PAS). We then invited two independent pathologists who were blinded to this study to identify the inflammatory scores based on the evaluation criteria described by Singh et al. [15].

### Autophagic flux assessment by Tandem-tagged mCherry-GFP-LC3B retrovirus generation

Generation of retroviruses encoding tandem-tagged mCherry-GFP-LC3B were prepared as previously described [16]. Briefly, NCM460 cells were seeded at 50% confluence into a 24-well plate and transfected with 1 ml Ad-mCherry-GFP-LC3 (Beyotime: C3011) overnight. Next, the cell culture medium was removed and replaced with fresh retrovirus-containing medium (MOI = 200) for 60 h. After incubation with oe-circGMCL1, si-circGMCL1, and blank for 48 h, cells were washed, fixed with 4% PFA, and then mounted with DAPI. Finally, fluorescence images were photographed under a confocal laser microscope (Leica SP8).

### RNA extraction and qRT-PCR

The nuclear and cytoplasmic fractions were extracted using NE-PER Nuclear and Cytoplasmic Extraction Reagents (Thermo Scientific). Total RNA was extracted using the TRIzol reagent (Invitrogen, Carlsbad, CA, USA) in accordance with the manufacturer’s instructions. RNA quality and quantity were determined using a Nanodrop and a bioanalyzer (Agilent Inc.). The qRT-PCR assay was performed using SYBGreen PCR system (Applied Biosystems, SA, USA). Relative circRNA and mRNA expressions were normalized to U6 and GAPDH, and calculated using the 2^−△△Ct^ method. All assays were performed in triplicate. Primer sequences are presented in Table 2.

**Table 2:** Primers sequences.

Name	Sequence (5′-3′)
Full-length sequencce of circGMCL1	GTGCCTTGAATGGCTTCTAAACAATTTGATGACTCACCAGAATGTTGAACT
	TTTTAAAGAACTCAGTATAAATGTCATGAAACAGCTCATTGGTTCATCTAAC
	TTATTTGTGATGCAAGTGGAGATGGATATATACACTGCTCTAAAAAAGTGGA
	TGTTCCTTCAACTTGTGCCTTCTTGGAATGGATCTTTAAAACAGCTTTTGAC
	AGAAACAGATGTCTGGTTTTCTAAACAGAGGAAAGATTTTGAAGGTATGGC
	CTTTCTTGAAACTGAACAAGGAAAACCATTTGTGTCAGTATTCAGACATTT
	AAGGTTACAATATATTATCAGTGATCTGGCTTCTGCAAGAATTATTGAACAA
	GATGCTGTAGTACCTTCAGAATGGCTCTCTTCTGTGTATAAACAGCAGTGGT
	TTGCTATGCTGCGGGCAGAACAGGACAGTGAGGTGGG
Human circGMCL1	
Forward	AGTACCTTCAGAATGGCTCTCT
Reverse	TTTAGAAGCCATTCAAGGCACC
Human miR-124-3p	
Forward	CATAAGGCACGCGGTGAATG
Reverse	GTCGTATCCAGTGCAGGGTC
RT Primer	GTCGTATCCAGTGCAGGGTCCGAGGTATTCGCACTGGA
	TACGACTTGGCA
miR-124-3p mimic	UAAGGCACGCGGUGAAUGCC
miR-124-3p inhibitor	GGCAUUCACCGCGUGCCUUA
Human ANXA7	
Forward	ACGTTTACTTGTGTCCATGTGC
Reverse	AGATTCATCGGTCCCTAGTCT
Human GMCL1	
Forward	CCACGGATTCTGTTACTGTGC
Reverse	TCCTCATCCTCGTCCGTCT
Human GAPDH	
Forward	TCCTGGGCTACACTGAGCAC
Reverse	CTGTTGCTGTAGCCAAATTCGTTG
Mouse circGMCL1	
Forward	ATTCTCTGGTACCTTCAGTGGA
Reverse	ACATCTGTTTCTGTCAAAAGCTG
Mouse miR-124-3p	
Forward	CCTAAGGCACGCGGTGAATG
Reverse	GTCGTATCCAGTGCAGGGTC
RT primer	GTCGTATCCAGTGCAGGGTCCGAGGTATTCGCA
	CTGGATACGACGGCATT
Mouse ANXA7	
Forward	CTTCGATGACTCAGGGCACC
Reverse	ACGGTTAGACACAACATCCACA
Mouse GMCL1	
Forward	CAGGTCGCATTTGGATCACT
Reverse	CGCACTGCTGTATCAAACCAT
Mouse GAPDH	
Forward	GTCAAGGCCGAGAATGGGAA
Reverse	CTCGTGGTTCACACCCATCA
U6	
Forward	ATTGGAACGATACAGAGAAGATT
Reverse	GGAACGCTTCACGAATTTG
Si-circGMCL1-1	Target: GTGAGGTGGGGTGCCTTGAAT
Sense	UCAAGGCACCCCACCUCAC
Antisense	GAGGUGGGGUGCCUUGAAU
Si-circGMCL1-2	Target:GTGGGGTGCCTTGAATGGCTT
Sense	GTGGGGTGCCTTGAATGGCTT
Antisense	GGGGUGCCUUGAAUGGCUU
Si-circGMCL1-3	Target: GCCTTGAATGGCTTCTAAACAAT
Sense	UGUUUAGAAGCCAUUCAAGGC
Antisense	CUUGAAUGGCUUCUAAACAAU
Oe-circGMCL1	-
Sense	GGAATTC TGAGGTGGGGTGCCTTGAAT
Antisense	CCCAAGCTT AGATCCATTCCAAGAAGGCACAA
circGMCL1 FISH probe	5′-FAM-AGCCATTCAAGGCACCCCACCTCACTGTCC-3′
miR-124-3p FISH probe	5′-CY3-TTGGCATTCACCGCGTGCCTTA-3′

*qRT-PCR* quantitative real time-polymerase chain reaction, *ANXA7* Annexin 7.

### Nucleic acid electrophoresis

Nucleic acid electrophoresis was performed as previously described [17]. The agarose gel electrophoresis method was used to analyze the cDNA and gDNA PCR products with TAE as the running buffer. Electrophoresis was conducted at 100 V for 30 min to separate DNA and the bands were observed under UV irradiation.

### Fluorescence in situ hybridization (FISH)

FISH assays were performed in colon tissues and NCM460 cells as previously described [18]. In brief, frozen tissues were fixed with 4% paraformaldehyde (DEPC, Servicebio) for 1–2 h, dehydrated in 15% sucrose solution for 8 h and in 30% sucrose solution overnight, fixed with 4% paraformaldehyde for 10 min. Cells were fixed with 4% paraformaldehyde for 20 min. Samples were then incubated with Proteinase K working solution (20 ug/ml) at 37 °C (15 min for tissues and 8 min for cells), and pre-hybridized by adding pre-hybridization solution at 37 °C for 1 h. Subsequently, sections were hybridized with FAM-conjugated circRNA probes (1 μM), CY3-conjugated miRNA probes (1 μM) overnight, and counterstained with DAPI after washing. Finally, slides were observed and photographed under the NIKON biological microscope (Nikon, Tokyo, Japan). Sequences for probes used are shown in Table 2.

### Luciferase reporter assay

WT and mutant 3’UTR sequences of circGMCL1 and ANXA7 were designed, synthesized, and inserted into vector GP-miRGLO (Genepharma, China). Cells were then co-transfected with a mixture of firefly and Renilla luciferase reporter, and miR-124-3p mimic or inhibitor. After incubation for 48 h, the relative luciferase activity was determined using a dual-luciferase reporter assay kit (Promega, Madison, WI, USA) following the manufacturer’s protocol.

### Statistical analyses

All statistical analyses were performed using SPSS 19.0 (SPSS, Chicago, IL, USA) and GraphPad Prism 8.0 (GraphPad, San Diego, CA, USA) software. Student’s *t* test, chi-square test, or one-way analysis of variance (ANOVA) test were used for data analysis as appropriate. The sample size was estimated using GPower software by calculating the power. Pearson’s correlation analysis was performed to evaluate the correlations among relative circGMCL1, miR-124-3p, and ANXA7 expressions, and inflammatory indices. A two-tailed *P* < 0.05 was considered statistically different.

## Results

### circRNAs screening and circGMCL1 verification

**Fig. 1:**
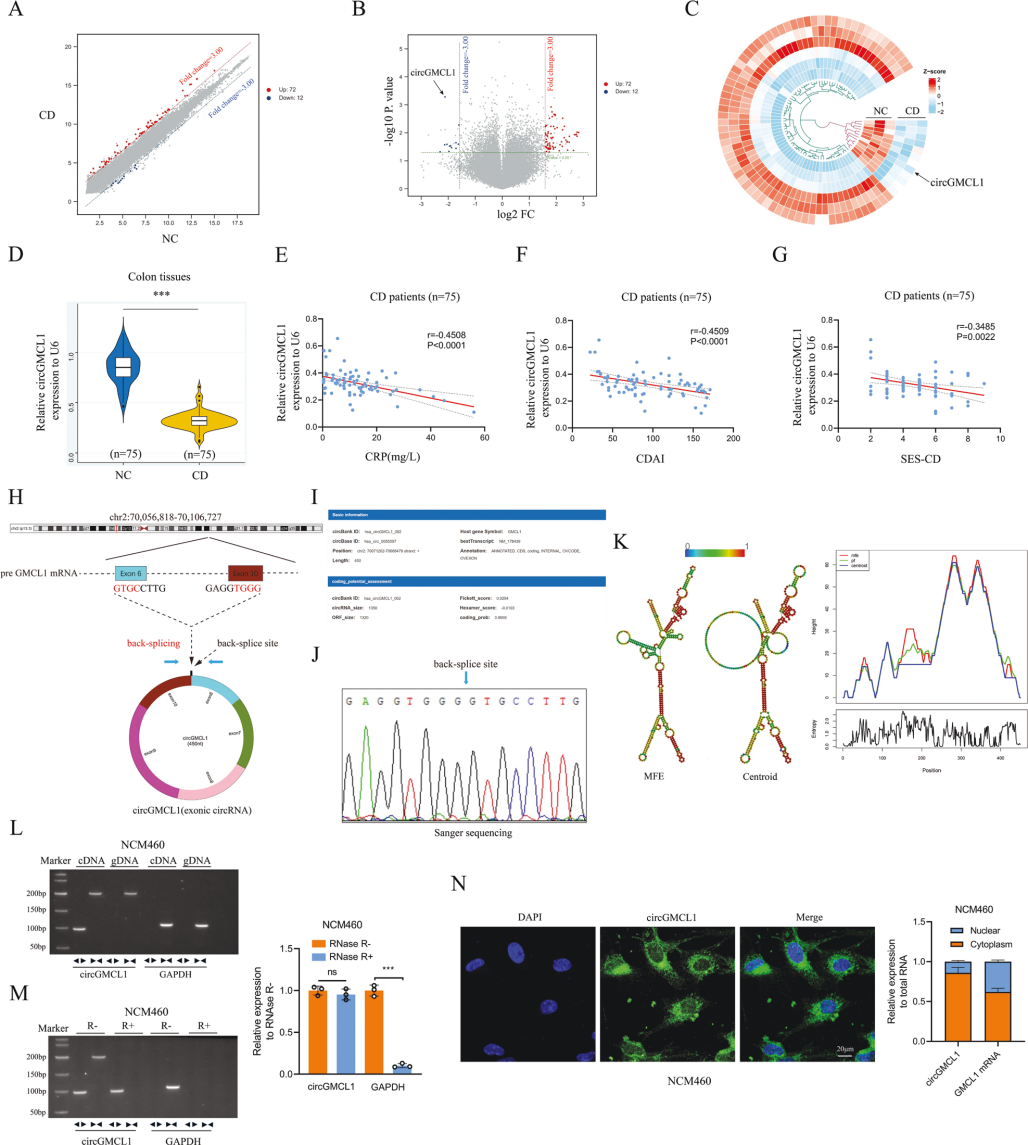
Identification of circGMCL1 as the candidate circRNA. Scatter plots **A** volcano plots **B** and heatmap **C** showing circRNA expression profile in colon tissues from CD patients and NCs (threshold: FC > 3, *P* < 0.05). **D** Relative circGMCL1 expression in colon tissues from CD patients and NCs as determined by qRT-PCR analysis. Relationship between circGMCL1 expression and CD-associated CRP **E**, CDAI **F** and SES-CD **G** in CD patients (*n* = 75). **H** The circle structure of circGMCL1 back-spliced by exon 6 and exon 10. **I** The basic information of circGMCL1. **J** The splicing of circGMCL1 by Sanger sequencing in NCM460 cell lines. **K** The predicted secondary structures of circGMCL1. **L** Nucleic acid electrophoresis for circGMCL1 in NCM460 cell lines by RT-PCR. **M** RNase R experiments by nucleic acid electrophoresis and qRT-PCR analyses. **N** FISH and qRT-PCR analyses performed to reveal the localization and expression of circGMCL1 in NCM460 cells. circRNA, circular RNA; CD, Crohn’s disease; NC, normal control; FC, fold change; qRT-PCR, quantitative real time-polymerase chain reaction; CRP, C-reactive protein; CDAI, Crohn’s disease activity index; SES-CD, simple endoscopic score for Crohn’s disease, MFE minimum free energy, FISH fluorescence in situ hybridization. Data are presented as mean ± SD. ***P < 0.001 by Student’s *t* tests.

We performed circRNA microarray using six colon samples (three CD vs three NC) to screen dysregulated circRNAs in CD. The circRNA expression profiles are presented by the scatter plot (Fig. 1A) and volcano plot (Fig. 1B). By setting FC > 3.0 and *P* < 0.05 as the threshold, we identified 84 differentially expressed circRNAs (72 upregulated and 12 downregulated) between CD patients and NCs (see the heatmap in Fig. 1C). Subsequently, the top three significantly dysregulated circRNAs were validated by qRT-PCR in an expanded sample size (75 CD vs 75 NC). The obtained results showed that hsa_circ_0055097 (circGMCL1) was the most significantly downregulated circRNA (Fig. 1D). Moreover, relative circGMCL1 expression had a moderately strong correlation with CRP (*r* = −0.4508, *P* < 0.001, Fig. 1E) and CDAI (*r* = −0.4509, *P* < 0.001, Fig. 1F), but had a poor positive correlation with SES-CD (*r* = −0.3485, *P* = 0.0022, Fig. 1G). These findings strongly suggest that circGMCL1, a specific circRNA, is closely associated with biochemical, clinical, and endoscopic characteristics of CD.

### Characterization of circGMCL1

Based on the database of University of California, Santa Cruz (UCSC) genome browser, and circBank, we generalize that circGMCL1 is derived from the GMCL1 gene and is located at chromosome 2 (70071262–70088479). It is formed via back-splicing of exon 6 and exon 10 (450 nt) (Fig. 1H), and has great protein coding potential (Fig. 1I). The results of Sanger sequencing using NCM460 cell line confirmed its circular structure (Fig. 1J). Figure 1K shows the minimum free energy (MFE) and centroid secondary structures of circGMCL1 predicted using RNAfold WebServer [19]. Furthermore, divergent and convergent primers were designed to amplify *circGMCL1* and *GMCL1* mRNAs. cDNA and gDNA analysis in NCM460 cell lines indicated that circGMCL1 was only amplified by divergent primers in cDNA (Fig. 1L). Moreover, RNase R experiment revealed that circGMCL1 was resistant to RNase R treatment (Fig. 1M). FISH analysis also showed that circGMCL1 was mainly localized in the cytoplasm (Fig. 1N), supporting its potential function as a ceRNA molecule.

### Identification of miR-124-3p as a target miRNA of circGMCL1

**Fig. 2:**
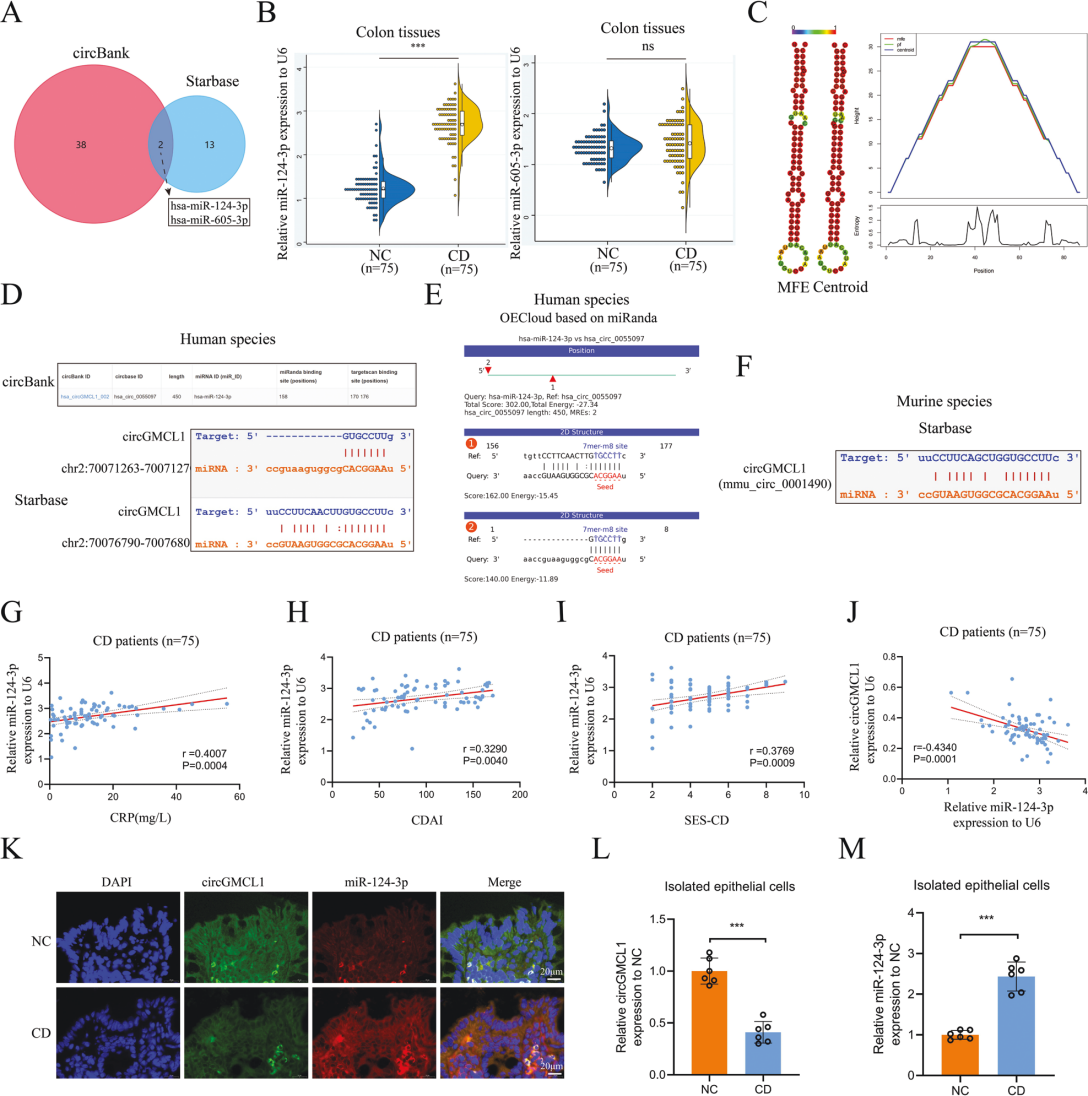
miR-124-3p was identified as one target of circGMCL1. **A** Venn diagram of target miRNAs established by overlapping circBank and starbase databases. **B** Relative miR-124-3p and miR-605-3p expression of CD patients and NCs was quantified by qRT-PCR test. **C** The predicted secondary structures of miR-124. The predicted binding sites between circGMCL1 and miR-124-3p in human species based on starbase and circBank databases (**D**), and OECloud based on miRanda (**E**). **F** The predicted binding sites between circGMCL1 and miR-124-3p in murine species based on the starbase. The linear correlation between relative miR-124-3p expression and CRP (**G**), CDAI (**H**), SES-CD (**I**), and circGMCL1 expression (**J**) in CD patients (*n* = 75) as determined by qRT-PCR test. **K** FISH analyses of circGMCL1 and miR-124-3p in colon tissues from CD and NCs. The relative expression of circGMCL1 (**L**) and miR-124-3p (**M**) in isolated epithelial cells from CD and NCs. MiRNA, microRNA, CD Crohn’s disease, NC normal control, qRT-PCR quantitative real time-polymerase chain reaction, CRP C-reactive protein, CDAI Crohn’s disease activity index, SES-CD simple endoscopic score for Crohn’s disease, MFE minimum free energy, FISH fluorescence in situ hybridization, ns, not significant. Data are expressed as mean ± SD. ***P < 0.001 by Student’s *t* tests.

Based on the ceRNA theory, we further explored the potential target miRNA of circGMCL1. The overlap of circBank and starbase databases indicated that miR-124-3p and miR-605-3p were the two potential target miRNAs (Fig. 2A). In addition, qRT-PCR analysis in colon tissues revealed that relative miR-124-3p expression was significantly higher in CD patients than in NCs (Fig. 2B). Thus, miR-124-3p was identified as the candidate target of circGMCL1. Figure 2C shows the predicted MFE and centroid secondary structures of miR-124, whereas Fig. 2D shows the binding sites predicted by circBank and starbase databases. The OECloud based on miRanda also predicted the binding site of miR-124-3p and circGMCL1 in human spicies (Fig. 2E), whereas the starbase database indicated the binding site of circGMCL1 and miR-124-3p in murine species (Fig. 2F). Moreover, although poor, a positive correlation was observed between relative miR-124-3p expression and inflammatory indices (CRP in Fig. 2G, CDAI in Fig. 2H, and SES-CD in Fig. 2I), suggesting that miR-124-3p is a potential CD-associated miRNA. Furthermore, the results obtained from FISH analyses indicated that the expressions of circGMCL1 and miR-124-3p showed a roughly negative correlation in colonic epithelial layer (Fig. 2K). Notably, the relative expression of circGMCL1 was significantly higher in epithelial cells isolated from CD patients compared to epithelial cells isolated from NCs (Fig. 2L), whereas the relative miR-124-3p expression showed a contrasting trend (Fig. 2M).

### Identification of Annexin 7 (ANXA7) as a target gene of miR-124-3p

**Fig. 3:**
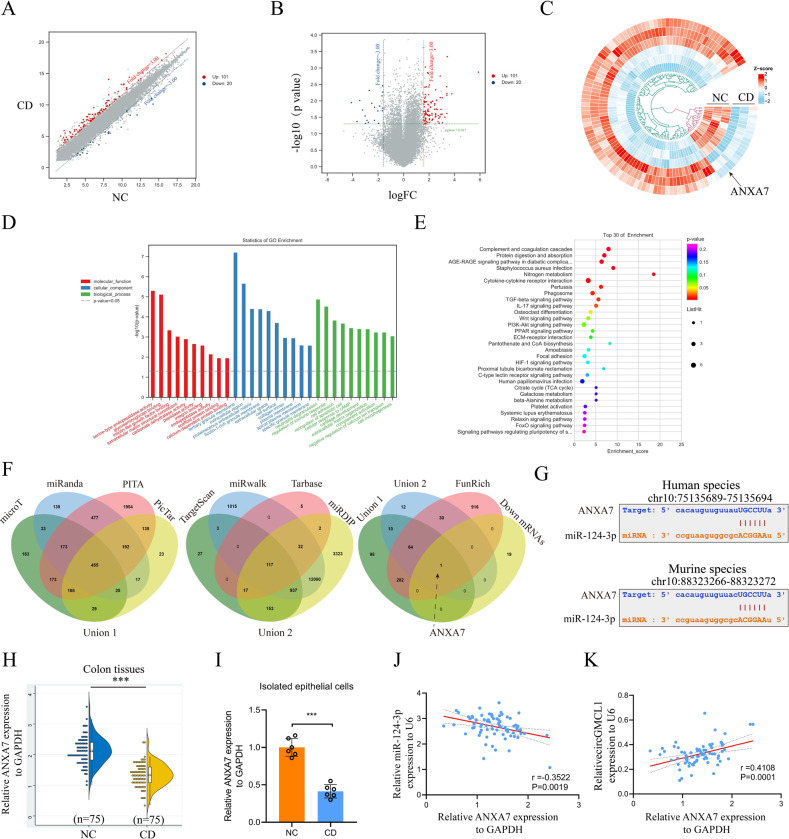
ANXA7 was identified as one target gene of miR-124-3p. Scatter plots (**A**), volcano plots (**B**), and heatmap (**C**) showing mRNA expression profile of colon tissues from CD patients and NCs (threshold: FC > 3, *P* < 0.05). The histogram (**D**) and bubble (**E**) displaying results of Go enrichment analyses, including BP, MF, and CC for the dysregulated mRNAs. Relative circGMCL1 expression in colon tissues from CD patients and NCs as determined by qRT-PCR analysis. **F** Venn diagram displaying the overlapping miR-124-3p target predicting algorithms and up-regulated mRNAs. **G** The predicted binding sites between miR-124-3p and ANXA7 in human and mouse species based on starbase database. Relative ANXA7 expression in colon tissues **H** and isolated epithelial cells **I** of CD patients and NCs as determined using qRT-PCR assay. The linear correlation between relative ANXA7 expression and miR-124-3p **J**, circGMCL1 **K** in CD patients (*n* = 75) as evaluated by qRT-PCR analysis. ANXA7 annexin 7, CD Crohn’s disease, NC normal control, FC fold change, GO gene ontology, BP biological processes, MF molecular function, CC cellular component, qRT-PCR quantitative real time-polymerase chain reaction. Data are presented as mean ± SD. ***P < 0.001 by Student’s *t* tests.

The mRNA expression profiles are presented by the scatter plot (Fig. 3A) and volcano plot (Fig. 3B). By setting FC > 3.0 and *P* < 0.05 as the threshold, we identified 121 differentially expressed circRNAs (101 upregulated and 20 downregulated) between CD patients and NCs (Fig. 3C). The GO enrichment analyses, including biological processes (BP), molecular function (MF), and cellular component (CC), are shown in Fig. 3D, E. Interactions between downregulated mRNAs, union 1 (microT, miRanda, miRmap, and PITA), union 2 (TargestScan, miRwalk, Tarbase, and miRDIP), and Funrich software revealed that *ANXA7* was the direct target gene of miR-124-3p. Figure 3G shows the predicted binding sites of miR-124-3p and ANXA7 in both human and mouse species. qRT-PCR analysis results showed that the relative *ANXA7* expression levels in colon tissues (Fig. 3H) and isolated epithelial cells (Fig. 3I) were both lower in CD patients than in NCs. Moreover, relative *ANXA7* expression exhibited a negative correlation with miR-124-3p (Fig. 3J) and a positive correlation with circGMCL1 (Fig. 3K) in CD patients. Collectively, these results strongly support the ceRNA theory of circGMCL1.

### Verification of the circGMCL1 ceRNA network

**Fig. 4:**
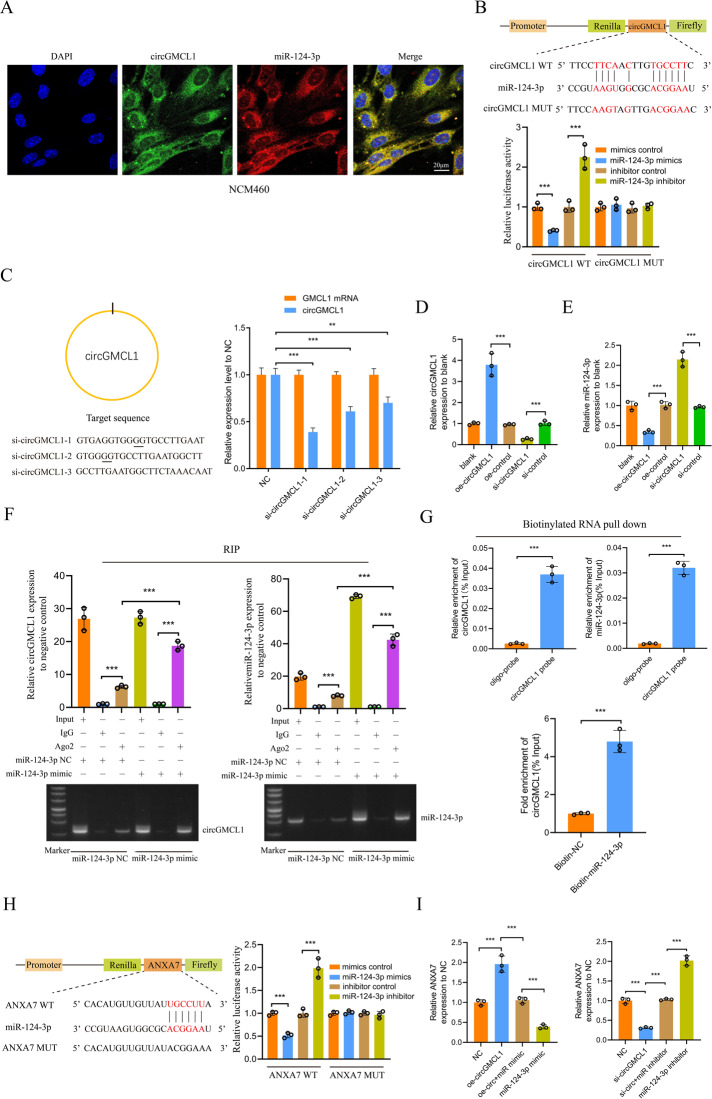
Verification of the circGMCL1 ceRNA network. **A** FISH analyses of circGMCL1 and miR-124-3p in NCM460 cells. **B** Luciferase reporter analysis showing the interaction between miR-124-3p and circGMCL1 in NCM460 cells. **C** Relative expression of circGMCL1 and GMCL1 in NCM460 cells transfected with three siRNAs as determined by qRT-PCR tests. Relative circGMCL1 (**D**) and miR-124-3p (**E**) expression in NCM460 cells transfected with control, oe-, si-circGMCL1 by qRT-PCR. **F** Nucleic acid electrophoresis and qRT-PCR analyses of circGMCL1 and miR-124-3p expression based on the Ago2 RIP assay. **G** Enrichment analysis of circGMCL1 and miR-124-3p after RNA pull down assay with circGMCL1 or miR-124-3p probe as evaluated by qRT-PCR assay. **H** Luciferase reporter analysis showing the interaction between miR-124-3p and ANXA7 in NCM460 cells. **I** Relative ANXA7 expression in NCM460 cells transfected with oe-, si-circGMCL1, miR-124-3p mimic, and inhibitor. FISH fluorescence in situ hybridization, WT wild-type; MUT, mutant; qRT-PCR, quantitative real time-polymerase chain reaction; RIP, RNA immunoprecipitation; ANXA7, annexin 7. Data are expressed as mean ± SD. **P < 0.01, ***P < 0.001 by Student’s t or one-way ANOVA test.

FISH analysis results indicated that circGMCL1 and miR-124-3p had consistent cellar localization in NCM460 cells (Fig. 4A). As shown in Fig. 4B, the relative luciferase reporter activity co-transfected with circGMCL1 WT was significantly high in miR-124-3p inhibitor group and low in miR-124-3p mimic group, indicating that circGMCL1 could directly bind to miR-124-3p. Next, we designed and synthesized three si-circGMCL1 sequences targeting three different positions. Results showed that the si-circGMCL1 sequence demonstrated the most significantly inhibitive effectiveness for circGMCL1 expressions (Fig. 4C). The qRT-PCR analysis verified the transfection efficacy of oe- or si-circGMCL1 in NCM460 cells (Fig. 4D). In addition, miR-124-3p expression in NCM460 cells was significantly downregulated by oe-circGMCL1, and significantly upregulated by si-circGMCL1 (Fig. 4E). To further explore the interactions between circGMCL1 and miR-124-3p, we performed Ago2 RIP experiments followed by agarose gel electrophoresis and qRT-PCR analyses. Figure 4F shows that circGMCL1 and miR-124-3p were both highly enriched in NCM460 cells transfected with miR-124-3p mimic compared to miR-124-3p mimic control. In addition, the biotinylated RNA pull down indicated that circGMCL1 and miR-124-3p were highly enriched in the circGMCL1 probe group (Fig. 4G). Similarly, circGMCL1 was enriched in the biotin-labeled miR-124-3p group (Fig. 4G). Furthermore, the luciferase reporter experiments indicated that miR-124-3p could directly bind to *ANXA7* (Fig. 4H). qRT-PCR analysis was then performed to verify the effects of circGMCL1 and miR-124-3p on *ANXA7* expressions. Results showed that the promotive (or inhibitive) effects of oe-circGMCL1 (or si-circGMCL1) on *ANXA7* expression could be rescued by miR-124-3p mimic (or miR-124-3p inhibitor) (Fig. 4I). Altogether, these results strongly validated the direct interactions among circGMCL1, miR-124-3p, and ANXA7.

### Biological functions of circGMCL1 and rescue experiments

**Fig. 5:**
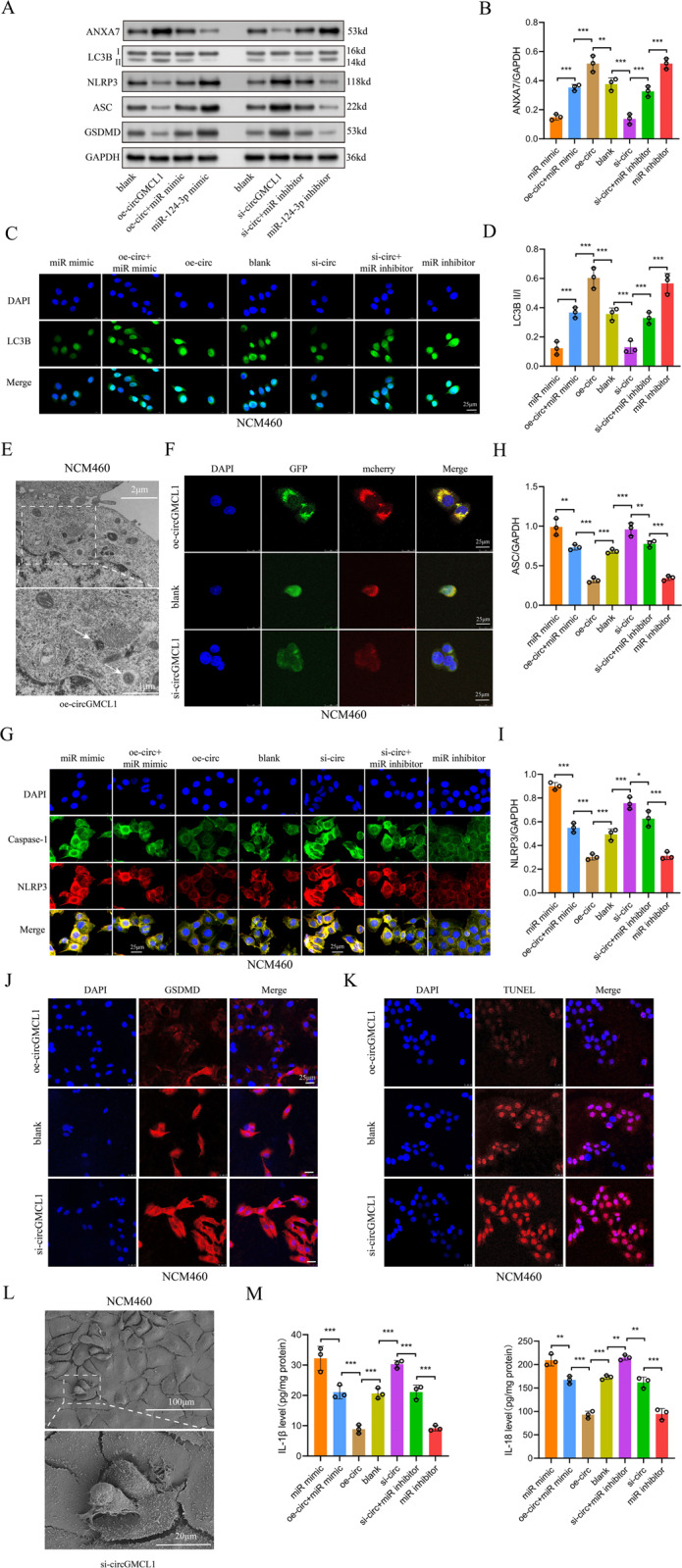
Biological functions of circGMCL1 in epithelial cells. **A** Results of western blotting showing protein levels of ANXA7, LC3B, NLRP3, ASC, and GSDMD. **B** Quantitative results of ANXA7 expression in western blotting test. Immunofluorescence (**C**) and quantitative analysis (**D**) of LC3B expression by western blotting in NCM460 cells. **E** Representative TEM images of the autophagosome in NCM460 cells transfected with oe-circcGMCL1. **F** Autophagy flux based on mCherry-GFP-LC3B retrovirus generation in NCM460 cells transfected with control, oe- and si-circGMCL1. **G** Immunofluorescence of NLRP3 and caspase-1 in NCM460 cells. Quantitative analysis of ASC **H** and NLRP3 **I** following western blotting test in NCM460 cells. GSDMD immunofluorescence (**J**) and TUNEL (**K**) assays in NCM460 cells. Representative pyroptosis SEM image (**L**). Proinflammatory cytokines (IL-1β and IL-18) in NCM460 cells **M**. QRT-PCR quantitative real time-polymerase chain reaction, ANXA7 annexin 7, TEM transmission electron microscope. Data are presented as mean ± SD. **P < 0.01, ***P < 0.001 by one-way ANOVA test.

A previous study reported that *ANXA7* is a crucial regulator of cell autophagy [20]. Considering that dysregulation of autophagy is implicated in CD pathogenesis [21, 22], this study focused on the biological function of circGMCL1 in CD via autophagy modulation. It is worth noting that NLRP3 inflammasome-mediated pyroptosis is closely associated with inflammatory response by producing IL-1β [23], and it can be modulated by cell autophagy [24, 25]. Therefore, we evaluated the biological functions of circGMCL1 in epithelial cells from the perspective of autophagy and NLRP3 inflammasome-mediated pyroptosis. The obtained results indicated that oe-circGMCL1 could significantly induce the protein expressions of ANXA7 and LC3B, and suppress expressions of NLRP3, ASC, and GSDMD (Fig. 5A). With GAPDH as the normal control, the quantification results of western blot indicated that ANXA7 protein expression was significantly upregulated by oe-circGMCL1 and significantly downregulated by si-circGMCL1 (Fig. 5B). The LC3B immunofluorescence (Fig. 5C) and LC3B II/I ratio by western blot (Fig. 5D) showed that oe-circGMCL1 could effectively induce cell autophagy. Figure 5E shows the representative TEM images of autophagosomes in oe-circGMCL1 transfected NCM460 cells. The autophagic flux assessment by mCherry-EGFP-LC3B retrovirus generation also indicated that oe-circGMCL1 could induce cell autophagy (Fig. 5F). The immunofluorescence (Fig. 5G) and western blot quantification (Fig. 5H, I) showed that NLRP3 inflammasome activity was suppressed by oe-circGMCL1, but the effect could be reversed by miR-124-3p mimic. The results of GSDMD immunofluorescence (Fig. 5J) and TUNEL (Fig. 5K) also indicated the suppressive functions of oe-circGMCL1 on epithelial pyroptosis. The representative pyroptosis SEM image in NCM460 cells transfected with si-circGMCL1 was displayed in Fig. 5L. Moreover, the proinflammatory cytokines (IL-1β and IL-18) in epithelial cells were significantly inhibited by oe-circGMCL1 (Fig. 5M).

### In vivo function of circGMCL1 in experimental colitis models

**Fig. 6:**
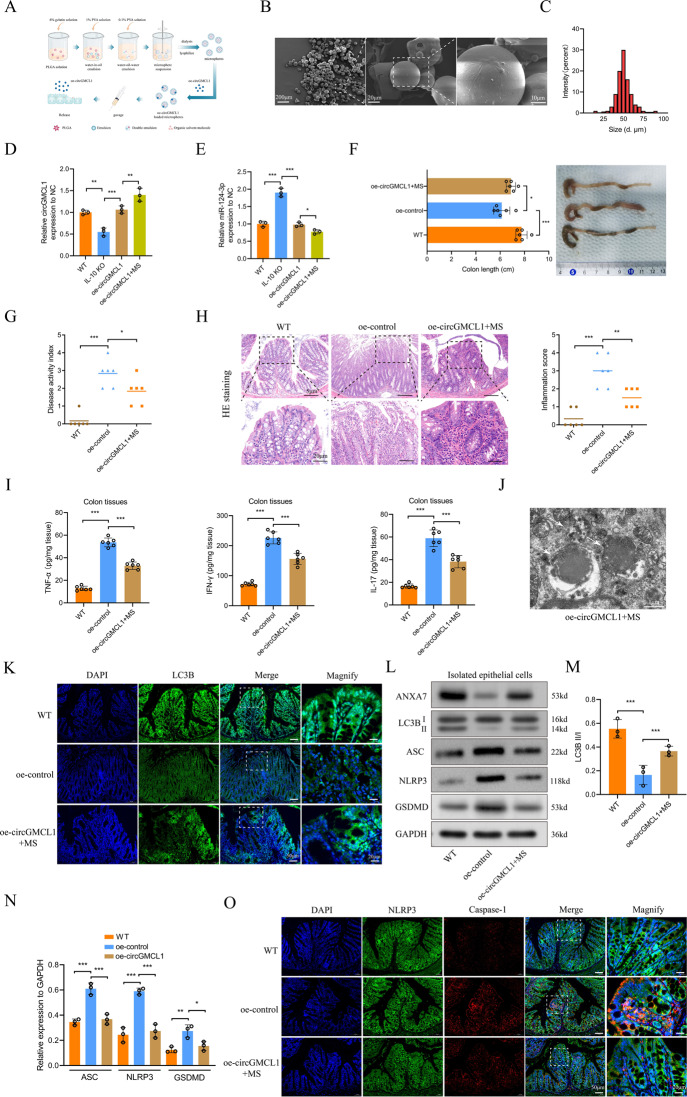
In vivo effects of circGMCL1 in IL-10 KO mice against colitis. **A** Preparation of PLGA MSs and the oe-circGMCL1 administration scheme. **B** Morphologies of PLGA MSs as observed under SEM. **C** Diameter distributions of MSs. Relative circGMCL1 **D** and miR-124-3p **E** expression in colon tissues treated with oe-circGMCL1. Colon length (**F**), DAI (**G**), pathological inflammatory infiltration **H**, proinflammatory cytokines (TNF-α, IFN-γ, and IL-17) in colon tissues **I** of IL-10 KO mice subjected to PLGA MS-carried oe-circGMCL1 treatment. **J** Representative TEM image of autophagosome in oe-circGMCL1-treated IL-10KO mice. **K** Immunofluorescence of LC3B in colon tissues. **L** Protein levels of ANXA7, LC3B, NLRP3, ASC, and GSDMD in isolated epithelial cells as quantified by western blotting. LC3B II/I ratio (**M**) and quantitative analysis of protein expression (**N**) by western blotting. (**O**) Immunofluorescence of NLRP3 and caspase-1 in colon tissues. PLGA poly(lactic-co-glycolic acid), MSs microspheres, qRT-PCR quantitative real time-polymerase chain reaction, SEM scanning electron microscope, TEM transmission electron microscope, DAI disease activity index, IL interleukin, IL-10 KO IL-10 knock-out, HE hematoxylin and eosin, TNF-α tumor necrosis factor-α, IFN-γ interferon-γ, ANXA7 annexin 7. Data are expressed as mean ± SD. *P < 0.05, **P < 0.01, ***P < 0.001 by one-way ANOVA test.

According to a previous study [26], PLGA MS delivery of medications can provide sustained release for longer duration, which significantly increases the potency of the medication with much smaller doses. Thus, we explored the in vivo functions of circGMCL1 using the prepared PLGA-MS delivery system (Fig. 6A). The surface morphologies SEM images of PLGA MSs are shown in Fig. 6B, whereas the diameter distribution of MSs, with a mean diameter of 40-70μm, is shown in Fig. 6C. The obtained results revealed that PLGA MSs-carried oe-circGMCL1 treatment could more effectively upregulate circGMCL1 (Fig. 6D) and downregulate the miR-124-3p expressions (Fig. 6E) in colon tissues of IL-10 KO mice compared to mice with oe-circGMCL1 treatment. Therefore, we chose the more effective PLGA MS-carried oe-circGMCL1 delivery to evaluate the in vivo functions. Results showed that the colonic shortening of IL-10 KO mice was significantly improved by PLGA MS-carried oe-circGMCL1 treatment (Fig. 6F). In addition, PLGA MS-carried oe-circGMCL1 treatment significantly alleviated the severity of colitis, which presented as decreased DAI (Fig. 6G), reduced inflammatory cell infiltration and inflammatory scores (Fig. 6H), and decreased colonic proinflammatory cytokines (TNF-α, IFN-γ, and IL-17) (Fig. 6I). The autophagy level was also significantly improved by oe-circGMCL1 treatment, which was shown by the representative autophagosome and autophagolysosome TEM images (Fig. 6J), and LC3B immunofluorescence (Fig. 6K). Western blot analysis demonstrated a significantly increased LC3B II/I ratio in epithelial cells isolated from oe-circGMCL1-treated IL-10 mice (Fig. 6L, M). Moreover, western blot analysis (Fig. 6L, N) and immunofluorescence (Fig. 6O) results indicated that NLRP3 inflammasome-mediated pyroptosis was significantly suppressed by oe-circGMCL1 treatment.

**Fig. 7:**
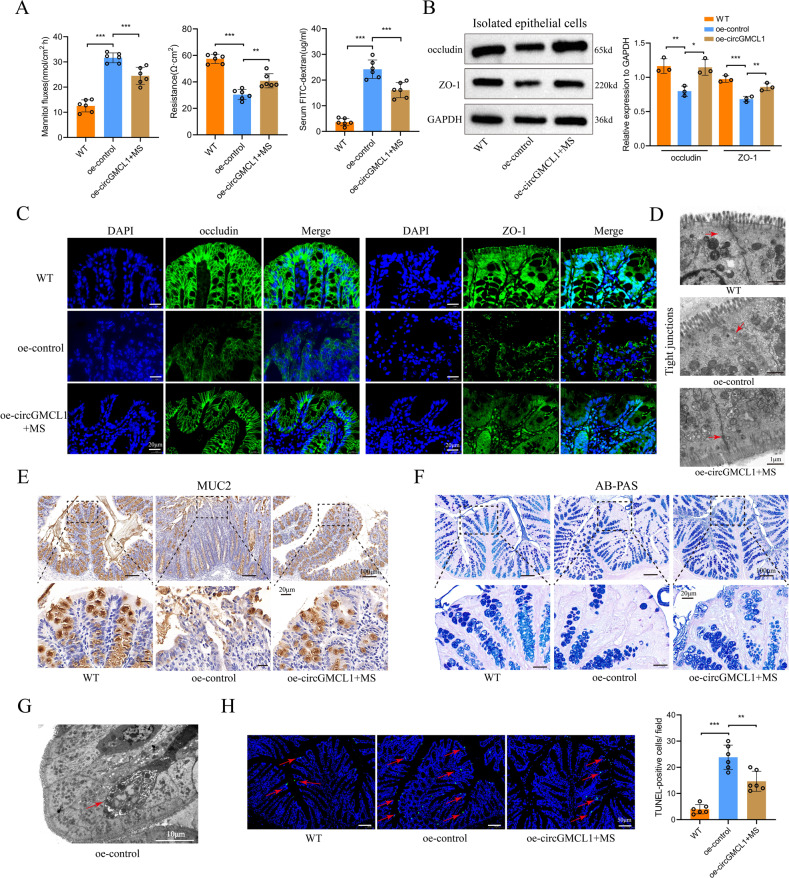
Impact of circGMCL1 on intestinal barrier function. **A** Intestinal permeability analyses. **B** Protein expression of TJ proteins (occluding and ZO-1) as determined by western blotting assay. **C** Immunofluorescence of TJ proteins. **D** Representative TEM images of TJs. **E** Immunohistochemical analysis of MUC2. **F** AB-PAS staining of colon sections. **G** Representative TEM images of apoptotic body in oe-control IL-10 KO mice. **H** Apoptosis of intestinal epithelial cells as evaluated using the TUNEL assay. TJ tight junction, TEM transmission electron microscope, FITC fluorescein isothiocyanate, TUNEL terminal deoxynucleotidyl transferase dUTP nick end labeling, MSs microspheres. Data are presented as mean ± SD. **P < 0.01, ***P < 0.001 by one-way ANOVA test.

Furthermore, we investigated the impacts of oe-circGMCL1 on the intestinal barrier function in experimental colitis models. Results obtained from the in vitro (mannitol fluxes and electronic resistance) and in vivo (FITC-dextran) experiments showed that the increased intestinal permeability was significantly improved by oe-circGMCL1 treatment (Fig. 7A). Western blot analysis results revealed that the protein expressions of TJ proteins (occludin and ZO-1) in isolated epithelial cells were significantly upregulated (Fig. 7B). In addition, immunofluorescence results showed that the distribution integrity (Fig. 7C) and morphology (Fig. 7D) of TJ proteins were improved by oe-circGMCL1 treatment. Results obtained after immumohistochemical staining of MUC2 (Fig. 7E) and AB-PAS staining (Fig. 7F) indicated that oe-circGMCL1 treatment could improve the intestinal mucus barrier function. Figure 7G shows the representative TEM image of apoptotic bodies of mice in oe-control group. TUNEL assay verified the inhibitive role of oe-circGMCL1 treatment for epithelial cells apoptosis (Fig. 7H). Overall, these results strongly indicate the protective role of circGMCL1 in intestinal barrier functions of experimental colitis models.

### Key findings

**Fig. 8:**
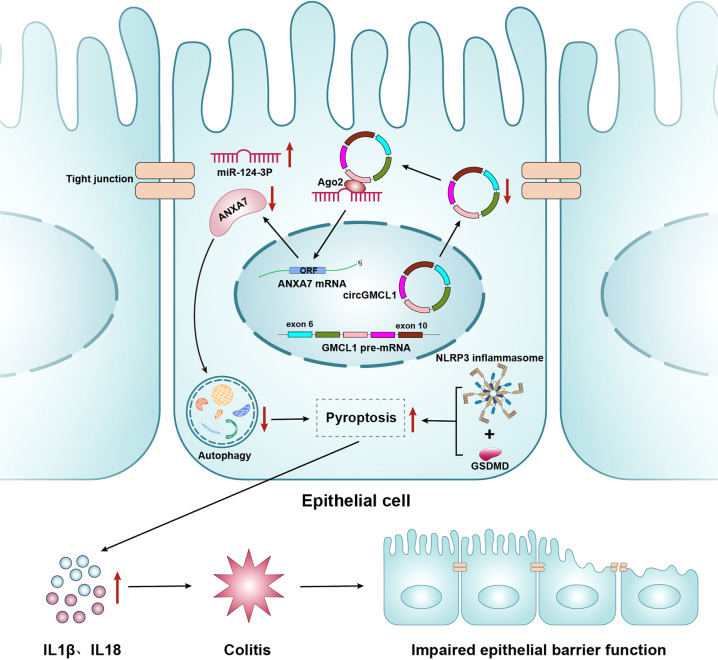
A schematic diagram of the biological function of circGMCL1 in Crohn’s colitis. The downregulation of circGMCL1 lowered ANXA7 expression by sponging miR-124-3p, thereby decreasing autophagy in epithelial cells and increasing NLRP3 inflammasome-mediated pyroptosis and release of proinflammatory cytokines. This resulted in aggravation of colitis and damage to the intestinal barrier function. ANXA7, annexin 7, ORF Open Reading Frame, IL interleukin.

This study has shown that downregulated circGMCL1 lowered ANXA7 expression through a ceRNA theory, thereby leading to autophagy deficiency in epithelial cells, followed by increased NLRP3 inflammasome-mediated pyroptosis and release of a large proportion of proinflammatory cytokines, which ultimately results in aggravated colitis and impaired intestinal barrier function (Fig. 8).

## Discussion

Although the association between circRNAs and CD has been the subject of increasing attention, functional and mechanical studies are quite limited. To the best of our knowledge, this study was the first to report that circGMCL1 is involved in CD pathogenesis through autophagy and pyroptosis modulation through regulating ANXA7 via sponging miR-124-3p. Moreover, the in vivo results suggest that circGMCL1 is a potential new biological therapeutic target for Crohn’s colitis.

Herein, microarray screening and further qRT-PCR analyses validated circGMCL1 as a CD-associated circRNA. Studies have shown that circRNAs can play important regulatory roles in various diseases by sponging miRNAs, interacting with RNA-binding proteins, and mediating transcription [27]. Among these mechanisms, the ceRNA hypothesis is the most widely reported and accepted mechanism of circRNAs [27]. Therefore, based on the ceRNA theory, we constructed a circGMCL1-miR-124-3p-ANXA7 ceRNA regulatory network through microarray and online prediction algorithms. The interactions among the ceRNA network were further validated by qRT-PCR, luciferase reporter, RNA pull down, and RIP assays. ANXA7 is a member of the annexin protein family, and is characterized by interaction with phospholipids in the presence of Ca^2+^ [28]. In addition, the annexin family plays critical roles in maintaining Ca^2+^ homeostasis, which is critical for autophagosome formation [29]. To date, it is not clear whether ANXA7 participates in CD. Wang et al. [28] reported that ANXA7 was an essential factor for LC3-II accumulation in human umbilical vein endothelial cells (HUVECs) and they identified 6-amino-2,3-dihydro-3-hydroxymethyl-1,4-benzoxazine (ABO) as an autophagy enhancer by targeting ANXA7. In addition, Meng et al. [20] demonstrated that ANXA7 can play a pivotal role in the process of mitophagy through interaction with BASP1. Our results also indicated the role of ANXA7 on epithelial cell autophagy, which is consistent with the conclusions of the above studies.

Autophagy, a physiological process of self-protection, may provide nutriment support to sustain cell survival and thus maintain stability in the intracellular environment [30]. Moreover, autophagy is an important self-regulation system of cells which is mediated by apoptosis [31]. Evidence shows that autophagy deficiency contributes to the pathogenesis of CD [32, 33]. Pyroptosis, a special form of programmed inflammatory cell rupture, plays a critical role in innate immune defense against bacterial [34]. A previous study reported that pyroptosis is involved in the pathogenesis of IBD [35]. Activation of inflammasome participates in pyroptosis, and the NLRP3 inflammasome regulates inflammatory responses [36]. It was found that NLRP3 inflammasome can initiate cell pyroptosis in combination with GSDMD, a molecule that stimulates pyroptosis by forming pores in the plasma membrane [37]. Furthermore, it has been reported that the induction of autophagy can suppress inflammation and pyroptosis [38, 39]. Thus, we explored the role of circGMCL1/miR-124-3p/DRAM1 network in colitis from the perspective of autophagy and NLRP3 inflammasome-induced pyroptosis. As expected, the results indicated that oe-circGMCL1 significantly suppressed NLRP3 inflammasome-induced pyroptosis and release of pro-inflammatory cytokines (IL-1β and IL-18) by inducing autophagy in intestinal epithelial cells.

To the best of our knowledge, this is the first study to highlight the biological functions, mechanisms, and therapeutic potential of circGMCL1 in Crohn’s colitis. Moreover, the interaction among circRNAs, cellular autophagy, and NLRP3 inflammasome-mediated pyroptosis is revealed in this study. However, some important issues should be addressed before circRNAs can become clinically applicable. For example, the best circRNA, target, dosage, administration strategy, and side effects need to be investigated. Moreover, this study focused on the NLRP3 inflammasome-mediated pyroptosis and autophagy in epithelial cells, whether other mechanisms are involved in this process, and whether the mechanism reported in this study applies to other cell types remain to be determined.

## Conclusion

In conclusion, this study suggests that circGMCL1 protects intestinal barrier function against Crohn’s colitis through alleviating NLRP3 inflammasome-mediated epithelial pyroptosis by promoting autophagy through regulating ANXA7 via sponging miR-124-3p.

## Supplementary information

aj-checklist

supplemental material

Full unedited gel and blots
