# Correction: PLGA-microspheres-carried circGMCL1 protects against Crohn’s colitis through alleviating NLRP3 inflammasome-induced pyroptosis by promoting autophagy

**DOI:** 10.1038/s41419-022-05266-x

**Published:** 2022-09-19

**Authors:** Jie Zhao, Ye Sun, Haojun Yang, Jun Qian, Yan Zhou, Yu Gong, Yi Dai, Yuwen Jiao, Weiming Zhu, Honggang Wang, Zhiliang Lin, Liming Tang

**Affiliations:** 1 Department of Gastrointestinal Surgery and Central Laboratory, The Affiliated Changzhou No. 2 People’s Hospital of Nanjing Medical University, Changzhou, China; 2 Department of Orthopaedics, The First Affiliated Hospital of Nanjing Medical University, Nanjing, China; 3 Department of Gastrointestinal Surgery, The Affiliated Changzhou No. 2 People’s Hospital of Nanjing Medical University, Changzhou, China; 4 Department of General Surgery, Jinling Hospital, Medical School of Nanjing University, Nanjing, China; 5 Department of Colorectal Disease, Intestinal Microenvironment Treatment Center, Shanghai Tenth People’s Hospital, Tenth People’s Hospital of Tongji University, Shanghai, China; 6 Department of General Surgery, Taizhou People’s Hospital, Taizhou Clinical Medical School of Nanjing Medical University, Nanjing, China

**Keywords:** Crohn's disease, Crohn's disease

Correction to: *Cell Death and Disease* 10.1038/s41419-022-05226-5, published online 10 September 2022

The original version of this article unfortunately contained errors in the affiliations and the order of authorship. The author institutions for the authors (Honggang Wang and Zhiliang Lin) were wrong (the opposite). In addition, the institution requested that the email address of the corresponding author (Liming Tang: tangliming@njmu.edu.cn) should rank the first.

