# Supplementary material for: PLGA-microspheres-carried circGMCL1 protects against Crohn’s colitis through alleviating NLRP3 inflammasome-induced pyroptosis by promoting autophagy
Source: Cell Death Dis. 2022 Sep 10;13(9):782. doi: 10.1038/s41419-022-05226-5 (PMC9464224; doi:10.1038/s41419-022-05226-5)
Supplement: Supplementary file 2 — supplemental material [file 41419_2022_5226_MOESM2_ESM.docx]

**Materials and methods**

**Microarray and bioinformatic analysis**

CircRNA/mRNA microarray analyses using six colon samples (three CD vs three NC) were conducted by OE Biotechnology Co., Ltd., (Shanghai, China) with the Agilent Human ceRNA MicroArray (Agilent Technologies, CA, USA). Briefly, total RNA was quantified by NanoDrop ND-2000 (Thermo Scientific), whereas RNA integrity was assessed by Agilent Bioanalyzer 2100 (Agilent Technologies). Notably, sample labeling and microarray hybridization were performed according to the manufacturer’s standard protocols. Raw data were obtained using Feature Extraction software (Agilent Technologies), followed by log2 transformation and normalization using the quantile algorithm. Bioinformatics analysis of the raw data was performed using the OECloud tools at <https://cloud.oebiotech.cn>. Differentially expressed genes (DEGs) were then identified with the thresholds of fold changes (FC) > 3.0 (or < -3.0) and p < 0.05. Next, gene ontology (GO) enrichment analyses, including biological processes (BP), molecular functions (MF), and cellular components (CC), were performed. The target mRNAs/miRNAs were identified by mRNA microarray and through bioinformatics analysis using several databases, including circBank (1), Starbase (2), microT (3), miRmap (<https://mirmap.ezlab.org/>), miRwalk (<http://mirwalk.umm.uni-heidelberg.de/>), Tarbase (4), miRDIP (5), miRanda (miranda.org), PITA (<https://omictools.com/pita-tool>), TargetScan (<http://www.targetscan.org/vert_80/>), and Funrich software (<http://www.funrich.org/>).

**Preparation of poly (lactic-co-glycolic acid)-microspheres (PLGA MSs)**

PLGA MSs encapsulating oe-circGMCL1 were prepared in accordance with the procedures described in Figure 6A. Briefly, PLGA MSs were obtained using a water-oil-water (W/O/W) double emulsion technique, followed by washing three times and freeze-drying. Scanning electron microscope (SEM) was used to assess the sizes and surface morphologies of the PLGA MSs. Oe-circGMCL1 was then added to the PLGA MSs at a weight ratio of 70:1 (MSs: oe-circGMCL1). For the treatment process, 3µg mixture of PLGA MSs carrying oe-circGMCL1 was administrated by gavage once a day for seven consecutive days.

**Measuring the levels of proinflammatory cytokines**

The levels of proinflammatory cytokines, including interleukin-1β (IL-1β), IL-17, IL-18, tumor necrosis factor-α (TNF-α), and interferon-γ (IFN-γ), in proximal colons or epithelial supernatants were measured using enzyme-linked immunosorbent assay (ELISA) (R&D Systems, Minneapolis, MN, USA). Briefly, colon tissues were rinsed, homogenized, and stored at ≤ -20℃ overnight. After two freeze-thaw cycles and centrifugation, the supernatants collected from homogenates were used for ELISA assays in accordance with the manufacturers’ instructions. The post intervention grouped NCM460 cells were seeded on a 24-well plate, followed by centrifugation and measurement of cytokines in the supernatants using ELISA assays.

**Analysis of epithelial apoptosis**

To assess epithelial apoptosis, terminal deoxynucleotidyl transferase (dUTP) nick end labeling (TUNEL) assay using *In Situ* Cell Death Detection Kits (Roche, Basel, Switzerland) was performed according to a previously described method (6). In summary, colon sections were permeabilized, washed, stained, and counterstained with 40,6-diamidino-2-phenylindole (DAPI; Servicebio, Shanghai, China). Next, sections were washed and mounted in 50% glycerol. Images were then captured using a confocal microscope (Olympus, Tokyo, Japan) and TUNEL positive cells/field were manually counted.

**Immunofluorescence staining**

The immunofluorescence staining of colon tissues was performed as previously described (7). Briefly, sections were fixed, washed, blocked with 5% normal goat serum, and then incubated with primary antibodies at 4℃ overnight. On the next day, sections were washed and then incubated with corresponding secondary antibodies (Cell Signaling Technology, MA, USA) at room temperature for 1 h. After nuclear counterstaining with DAPI, a confocal microscope (Olympus, Tokyo, Japan) was used to visualize the samples. MUC2 immunohistochemistry was also performed to assess the colonic mucous barrier. Primary antibodies used in this analysis were: anti-LC3 (Abcam: ab192890, Cambridge, UK), anti-Occludin (Abcam: ab216327), anti-ZO-1 (Abcam: ab221547), anti-NLRP3 (Abcam: ab4207), Anti-Caspase 1 (Sigma: AB1871), and anti-MUC2 (Novus Biologicals: NBP1-31231).

**Isolation of** **primary epithelial cells**

Primary epithelial cells were isolated from human colon samples in accordance with a previously described protocol (8). Briefly, colon samples from the sacrificed mice were cut into pieces (0.5 cm) and placed in cold PBS to remove debris. In addition, colonic samples were obtained from CD patients or NC controls during operation or endoscopic examination. To isolate primary epithelial cells, samples were incubated in PBS with 2mmol/l DTT and 1mmol/l EDTA under gentle shaking (37°C, 20 × 2 min). Cells were further purified by density gradient centrifugation with 20% and 40% percoll-RPMI solution. After centrifugation at 200 g for 5 min, the isolated epithelium was collected for further experiments.

**Cell immunoﬂuorescence analysis**

The immunofluorescence analysis of NCM460 cells was performed as previously described (9). Briefly, NCM460 cells were fixed, permeabilized, blocked, and incubated with primary antibodies (4°C, overnight). After washing, cells were incubated with appropriate secondary antibodies, followed by nuclear counterstaining with DAPI. Finally, visualization was performed using a confocal microscope (Carl Zeiss, Oberkochen, Germany).

**Transmission electron microscopy (TEM)**

TEM was used to assess the morphologies and locations of TJs, as well as autophagosome or autophagolysosome according to a previously described protocol (10). Briefly, sections were fixed, cut into small pieces, post-fixed, dehydrated, infiltrated in Epon812, and embedded in resin. After staining with uranyl acetate and lead citrate, the slices were visualized and photographed using Hitachi H-600 TEM.

**Intestinal permeability assay**

The *in vitro* permeability of colon segments was assessed by Ussing chamber assays as previously described (11). In brief, the serosal surfaces of mucosa were exposed to Ringer’s buffer, and maintained at 37℃. Basal mannitol fluxes were determined by adding mannitol onto the mucosal side. Electronic resistance was calculated with transepithelial potential difference (PD) and short circuit current (Isc) values based on Ohm’s law (12). The *in vivo* intestinal permeability assay was assessed using Fluorescein isothiocyanate (FITC)–dextran (Sigma-Aldrich) as previously described (13). FITC-dextran (60mg/100 g body weigh) was then administrated intragastrically. After 4 h, serum expression of dextran was determined using a fluorescence microplate reader (BMG Labtech, Ortenberg, Germany).

**Western blot analysis**

Protein expressions were determined by western blot analysis as previously described (14). Briefly, proteins were extracted from colon tissues or cells using Radio Immunoprecipitation Assay lysis buffer (RIPA, Beyotime, China). A bicinchoninic acid (BCA) Protein Assay Reagent Kit (Pierce Biotechnology, Rockford, IL, USA) was used to determine the protein levels. Proteins were then resolved using 10% sodium dodecyl sulfate polyacrylamide gel electrophoresis (SDS-PAGE), and transferred to a polyvinylidene fluoride (PVDF) membrane (Millipore, MA, USA). After blocking with 5% bovine serum albumin (BSA), membranes were incubated with primary antibodies (4°C, overnight). On the next day, membranes were washed and then incubated with corresponding secondary antibodies (room temperature, 2 h). Finally, blots were visualized with the ECL substrate and the greyscale values were analyzed using ImageJ (Media Cybernetics, Silver Spring, MD, USA), with GAPDH as the normal control. Primary antibodies used in this analysis were: anti-LC3 (Abcam: ab192890), anti-Occludin (Abcam: ab216327), anti-ZO-1 (Abcam: ab221547), anti-NLRP3 (Abcam: ab4207), anti-ASC (Abcam: ab175449), and anti-GSDMD (Abcam: ab219800, ab155233).

**RNase R treatment**

The RNA (10μg) extracted from NCM460 cells was incubated with RNase R (3 U/μg, Epicenter, Sweden) at 37°C for 30 min. RNA samples were then reverse transcribed using divergent and convergent primers. Next, qRT-PCR and nucleic acid electrophoresis were performed.

**RNA immunoprecipitation (RIP) assay**

As previously described (15), RIP assay was performed using Magna RIP™ RNA-binding protein IP kit (Millipore, USA) following the manufacturer’s instructions. After transfection with miR-124-3p mimics or control, NCM460 cells were lysed and cell extracts were obtained. Cells were then incubated with magnetic beads conjugated with anti-Argonaute 2 (Ago2) or anti-IgG antibodies (Millipore, USA), followed by removal of proteins and incubation with Proteinase K (Servicebio). Finally, RNA was extracted using TRIzol Reagent (Invitrogen) for further agarose gel electrophoresis and qRT-PCR analyses.

**Biotin-coupled probe RNA pull down assay**

Biotinylated circGMCL1 and miR-124-3p pull down assays were performed as described in a previous study (16). Briefly, 1 × 10^7^ NCM460 cells were harvested, lysed, and then sonicated. Next, probe-coated beads were generated by incubating probes using streptavidin dynabeads (Invitrogen, USA). After incubation with probe-coated beads at 4°C overnight, cell lysates were washed and the RNA complexes were eluted. The obtained purified RNAs were then used for further qRT-PCR analyses with Trizol Reagent (Takara, Japan).

**Oligonucleotide transfection**

Before transfection, NCM460 cells were incubated with 10 ng/ml LPS for 3 h, followed by 5 mM ATP for 30 min (InvivoGen, San Diego, CA) to activate the NLRP3 inflammasome. NCM460 cells were then seeded into 6-well plates and incubated to 60–70% confluence. CircGMCL1 siRNA, miR-124-3p mimic, inhibitor, and negative control oligonucleotides (Geneseed, Guangzhou, China) were transfected using Lipofectamine™2000 reagent (Invitrogen, USA).

**Vector construction and transfection**

We synthesized oe-circGMCL1 sequence and cloned it into the circRNA overexpression vector pcDNA3.1 (GenePharma, Shanghai, China), with an empty vector as the negative control. The stable overexpression transfection of circGMCL1 was performed in accordance with a previously described method (17). The primer was shown in Table 2.

**References**

1. Liu M, Wang Q, Shen J, Yang BB, and Ding X. Circbank: a comprehensive database for circRNA with standard nomenclature. *RNA Biol.* 2019;16(7):899-905.

2. Li JH, Liu S, Zhou H, Qu LH, and Yang JH. starBase v2.0: decoding miRNA-ceRNA, miRNA-ncRNA and protein-RNA interaction networks from large-scale CLIP-Seq data. *Nucleic Acids Res.* 2014;42(Database issue):D92-7.

3. Paraskevopoulou MD, Georgakilas G, Kostoulas N, Vlachos IS, Vergoulis T, Reczko M, et al. DIANA-microT web server v5.0: service integration into miRNA functional analysis workflows. *Nucleic Acids Res.* 2013;41(Web Server issue):W169-73.

4. Karagkouni D, Paraskevopoulou MD, Chatzopoulos S, Vlachos IS, Tastsoglou S, Kanellos I, et al. DIANA-TarBase v8: a decade-long collection of experimentally supported miRNA-gene interactions. *Nucleic Acids Res.* 2018;46(D1):D239-D45.

5. Tokar T, Pastrello C, Rossos AEM, Abovsky M, Hauschild AC, Tsay M, et al. mirDIP 4.1-integrative database of human microRNA target predictions. *Nucleic Acids Res.* 2018;46(D1):D360-D70.

6. Zhao J, Wang H, Yang H, Zhou Y, and Tang L. Autophagy induction by rapamycin ameliorates experimental colitis and improves intestinal epithelial barrier function in IL-10 knockout mice. *Int Immunopharmacol.* 2020;81:105977.

7. Clayburgh DR, Barrett TA, Tang Y, Meddings JB, Van Eldik LJ, Watterson DM, et al. Epithelial myosin light chain kinase-dependent barrier dysfunction mediates T cell activation-induced diarrhea in vivo. *J Clin Invest.* 2005;115(10):2702-15.

8. Yu M, Luo Y, Cong Z, Mu Y, Qiu Y, and Zhong M. MicroRNA-590-5p Inhibits Intestinal Inflammation by Targeting YAP. *J Crohns Colitis.* 2018;12(8):993-1004.

9. Nguyen HT, Dalmasso G, Muller S, Carriere J, Seibold F, and Darfeuille-Michaud A. Crohn's disease-associated adherent invasive Escherichia coli modulate levels of microRNAs in intestinal epithelial cells to reduce autophagy. *Gastroenterology.* 2014;146(2):508-19.

10. Wang H, Dong J, Shi P, Liu J, Zuo L, Li Y, et al. Anti-mouse CD52 monoclonal antibody ameliorates intestinal epithelial barrier function in interleukin-10 knockout mice with spontaneous chronic colitis. *Immunology.* 2015;144(2):254-62.

11. Arrieta MC, Madsen K, Doyle J, and Meddings J. Reducing small intestinal permeability attenuates colitis in the IL10 gene-deficient mouse. *Gut.* 2009;58(1):41-8.

12. Looijer-van Langen M, Hotte N, Dieleman LA, Albert E, Mulder C, and Madsen KL. Estrogen receptor-beta signaling modulates epithelial barrier function. *Am J Physiol Gastrointest Liver Physiol.* 2011;300(4):G621-6.

13. Ahmad R, Rah B, Bastola D, Dhawan P, and Singh AB. Obesity-induces Organ and Tissue Specific Tight Junction Restructuring and Barrier Deregulation by Claudin Switching. *Sci Rep.* 2017;7(1):5125.

14. Ji ML, Jiang H, Zhang XJ, Shi PL, Li C, Wu H, et al. Preclinical development of a microRNA-based therapy for intervertebral disc degeneration. *Nat Commun.* 2018;9(1):5051.

15. Luo Z, Rong Z, Zhang J, Zhu Z, Yu Z, Li T, et al. Circular RNA circCCDC9 acts as a miR-6792-3p sponge to suppress the progression of gastric cancer through regulating CAV1 expression. *Mol Cancer.* 2020;19(1):86.

16. Du WW, Yang W, Liu E, Yang Z, Dhaliwal P, and Yang BB. Foxo3 circular RNA retards cell cycle progression via forming ternary complexes with p21 and CDK2. *Nucleic Acids Res.* 2016;44(6):2846-58.

17. Sang Y, Chen B, Song X, Li Y, Liang Y, Han D, et al. circRNA_0025202 Regulates Tamoxifen Sensitivity and Tumor Progression via Regulating the miR-182-5p/FOXO3a Axis in Breast Cancer. *Mol Ther.* 2019;27(9):1638-52.
