# Supplementary material for: PLGA-microspheres-carried circGMCL1 protects against Crohn’s colitis through alleviating NLRP3 inflammasome-induced pyroptosis by promoting autophagy
Source: Cell Death Dis. 2022 Sep 10;13(9):782. doi: 10.1038/s41419-022-05226-5 (PMC9464224; doi:10.1038/s41419-022-05226-5)
Supplement: Supplementary file 3 — Full unedited gel and blots [file 41419_2022_5226_MOESM3_ESM.docx]

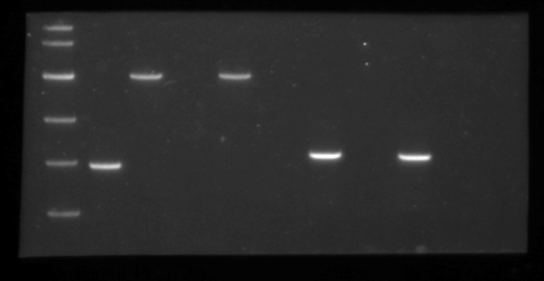


Full unedited gel for Figure 1L


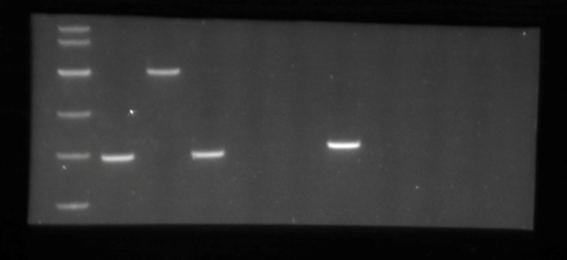


Full unedited gel for Figure 1M


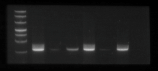


Full unedited gel for Figure 4F1


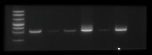


Full unedited gel for Figure 4F2





Full unedited gel for Figure 5A (ANXA7)





Full unedited gel for Figure 5A (ASC)





Full unedited gel for Figure 5A (GAPDH)





Full unedited gel for Figure 5A (LC3B)





Full unedited gel for Figure 5A (GSDMD)





Full unedited gel for Figure 5A (NLRP3)


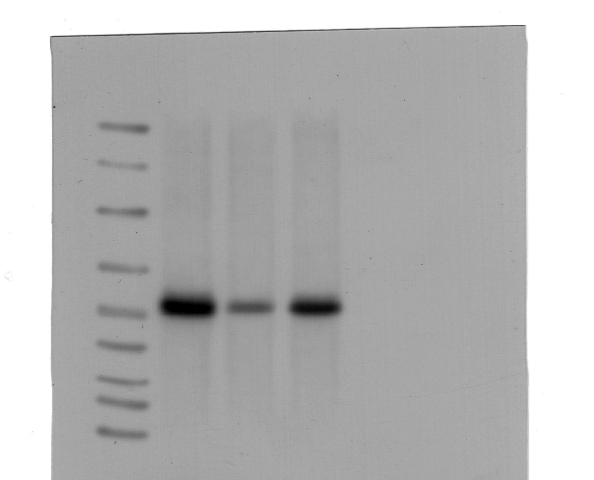


Full unedited gel for Figure 6L (ANXA7)


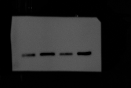


Full unedited gel for Figure 6L (ASC)


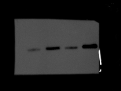


Full unedited gel for Figure 6L (NLRP3)





Full unedited gel for Figure 6L (GSDMD)





Full unedited gel for Figure 6L (LC3B)





Full unedited gel for Figure 6L (GAPDH)


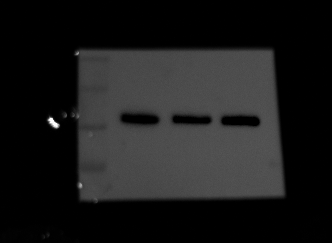


Full unedited gel for Figure 7B (GAPDH)


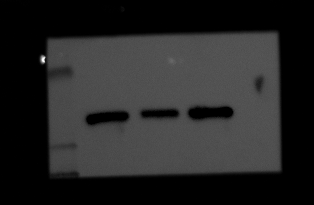


Full unedited gel for Figure 7B (occludin)


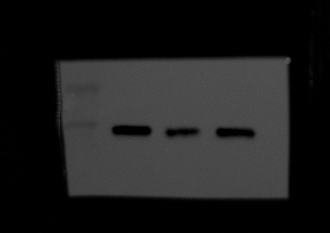


Full unedited gel for Figure 7B (ZO-1)
